# The Tight-Junction Protein Claudin-6 Induces Epithelial Differentiation from Mouse F9 and Embryonic Stem Cells

**DOI:** 10.1371/journal.pone.0075106

**Published:** 2013-10-08

**Authors:** Kotaro Sugimoto, Naoki Ichikawa-Tomikawa, Seiro Satohisa, Yushi Akashi, Risa Kanai, Tsuyoshi Saito, Norimasa Sawada, Hideki Chiba

**Affiliations:** 1 Department of Pathology, Sapporo Medical University School of Medicine, Sapporo, Japan; 2 Department of Obstetrics and Gynecology, Sapporo Medical University School of Medicine, Sapporo, Japan; 3 Department of Basic Pathology, Fukushima Medical University School of Medicine, Fukushima, Japan; University of Chicago, United States of America

## Abstract

During epithelialization, cell adhesions and polarity must be established to maintain tissue assemblies and separate the biological compartments in the body. However, the molecular basis of epithelial morphogenesis, in particular, a role of cell adhesion molecules in epithelial differentiation from stem cells, remains unclear. Here, we show that the stable and conditional expression of a tight-junction protein, claudin-6 (Cldn6), triggers epithelial morphogenesis in mouse F9 stem cells. We also demonstrate that Cldn6 induces the expression of other tight-junction and microvillus molecules including Cldn7, occludin, ZO-1α+, and ezrin/radixin/moesin-binding phosphoprotein50. These events were inhibited by attenuation of Cldn6 using RNA interference or the C-terminal half of *Clostridium Perfringens* enterotoxin. Furthermore, similar results were obtained in mouse embryonic stem cells. Thus, we have uncovered that the Cldn6 functions as a novel cue to induce epithelial differentiation.

## Introduction

Stem cell maintenance, self-renewal, and differentiation are regulated by both intrinsic and extrinsic cues [1]. Among intrinsic signals, there is accumulating evidence that specific transcription factors induce stem cell fate [2]–[4]. Extrinsic cues, such as a wide range of growth factors and small molecules, as well as cell-cell and cell-matrix adhesion, also influence stem cell behavior [4]–[7]. Concerning the cell-cell contact, DE-cadherin-medicated adhesion is essential for holding *Drosophila* germ stem cells in their niche and for their maintenance [8], [9]. In addition, the cell-adhesion function of β-catenin is required for definitive endoderm formation and neuronal differentiation in mouse embryonic stem cells [10]. However, it is largely unknown whether and how cell adhesion molecules control stem cell fate.

Mature epithelial cells are connected by apical junctional complexes (AJCs) that consist of tight junctions, adherence junctions and desmosomes, and exhibit apicobasal cell polarity [11]–[13]. On the other hand, mouse F9 stem cells show very little spontaneous differentiation, but differentiate upon retinoic acid treatment or under particular culture conditions into primitive and visceral endoderm-like cells, both of which represent matured columnar epithelia [14]. Hence, they provide an attractive system to investigate the molecular mechanism underlying epithelial morphogenesis. We previously established the cell line F9:rtTA:Cre-ER^T^ L32T2 (also called F9 L32T2), which allows Tet-on inducible gene expression and tamoxifen-dependent Cre-mediated recombination without altering its general characteristics [15], and demonstrated that two members of the nuclear receptor superfamily, retinoid receptors and hepatocyte nuclear factor 4α (HNF4α), triggered the formation of cell-cell junctions and epithelial polarity [16]–[19].

Claudins (Cldns) are essential components of tight junctions, the apical-most constituents of AJCs [20]–[24]. Among the 27 members of the Cldn family, Cldn6 is not expressed in adult differentiated cells of any organ except for renal podocytes [25] but expressed in various types of embryonic epithelia [26], [27]. Taken together with our previous finding that Cldn6 is rapidly and intensively expressed during the epithelial differentiation processes of F9 cells [16], [17], we hypothesized that Cldn6-dependent cell adhesion induced epithelial morphogenesis. In this study, we show, by using mouse F9 and embryonal stem cells, that Cldn6 can indeed act as a cue to trigger epithelial differentiation from stem cells.

## Results and Discussion

### Cldn6 Provokes Epithelial Differentiation in F9 Stem Cells

**Figure 1:**
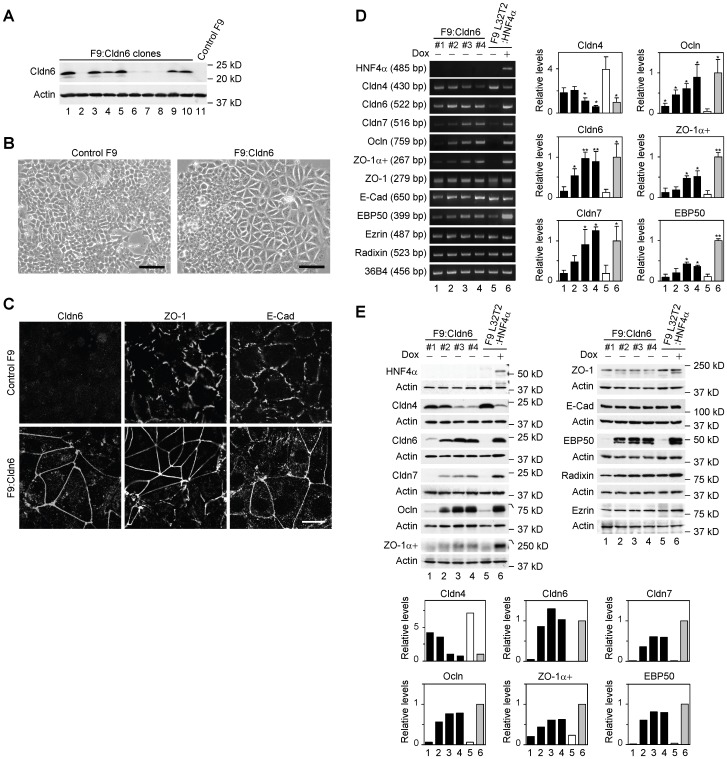
Cldn6 triggers epithelial differentiation in mouse F9 stem cells. (A) Western blot showing expression of Cldn6 protein in 10 clones of F9:Cldn6 cells and control F9 cells. (B and C) Morphological appearance and localization of Cldn6, ZO-1 and E-cadherin (E-Cad) in control F9 and F9:Cldn6 cells. Scale bars, (A) 50 µm; (B) 20 µm. (D) RT-PCR for the indicated molecules in 4 clones of F9:Cldn6 cells and F9 L32T2:HNF4α cells treated for 72 h with the vehicle or 1.0 µg/ml doxycycline (Dox). Quantification of the mRNA levels is shown in the histograms (mean+SD; *n* = 4). *P<0.05; **P<0.001 compared with values of the lane 5. (E) Western blot for the indicated molecules in F9:Cldn6 cells and F9 L32T2:HNF4α cells treated for 72 h with the vehicle or 1.0 µg/ml Dox. Quantification of the protein levels is shown in the histograms.

**Figure 2:**
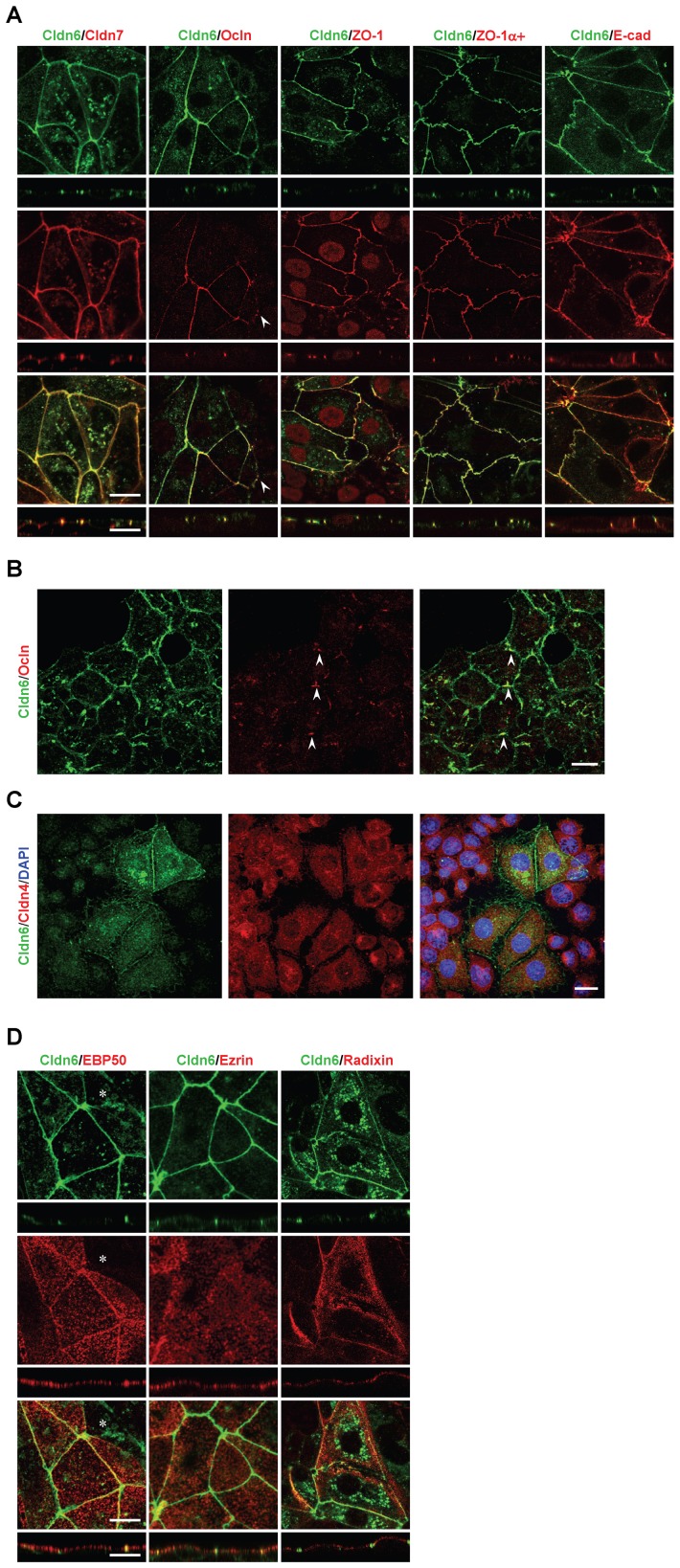
Cldn6 induces the formation of cell-cell junctions and apicobasal cell polarity in F9 stem cells. (A and D) X-Y and Z projections of F9:Cldn6 cells stained for Cldn6 together with the tight-junction markers (Cldn7, Ocln, ZO-1, and ZO-1α+ variant), the basolateral maker (E-Cad), and the apical markers (ezrin, radixin, and EBP50). The arrowhead indicates the recruitment of Ocln to a part of Cldn6-positive premmature cell-cell junctions. The asterisk shows the absence of EBP50 on the apical surfaces of undifferentiated cells possessing Cldn6-positive premature cell-cell junctions. Scale bars, 20 µm. (B) Confocal images of undifferentiated areas of F9:Cldn6 cells stained for Cldn6 together with Ocln. The arrowheads indicate the partial recruitment of Ocln to Cldn6-positive cell borders of undifferentiated cells. Scale bar, 20 µm. (C) Confocal images of F9:Cldn6 cells stained for Cldn6 together with Cldn4 and DAPI. Scale bar, 20 µm.

**Figure 3:**
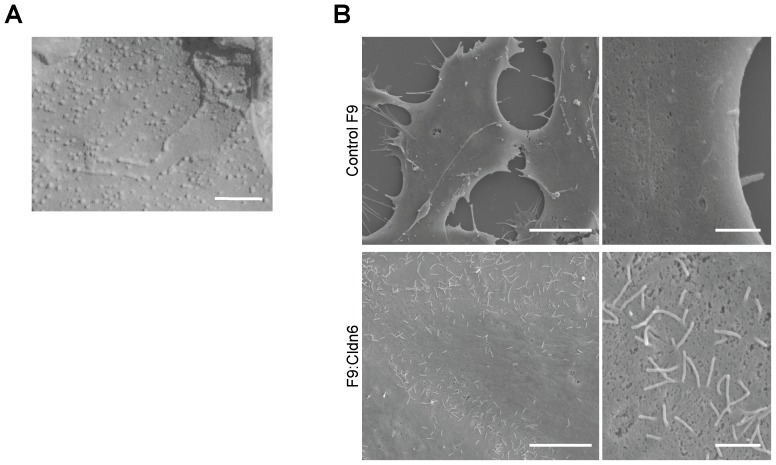
Cldn6 induces the formation of tight-junction strands and microvilli in F9 stem cells. (A) Freeze-fracture EM micrograph of F9:Cldn6 cells. Scale bar, 100 nm. (B) SEM micrographs of control F9 and F9:Cldn6 cells. Scale bars, left 5 µm; right 1 µm.

To verify the involvement of Cldn6 in epithelial differentiation, we first established F9:Cldn6 cells that stably expressed Cldn6 (Figure 1A). By phase-contrast microscopic analysis, approximately 30% of areas of F9:Cldn6 clones 3 and 4, which strongly expressed Cldn6, became large and polygonal in shape after 96 h after passage (Figure 1B, 1E). We subsequently examined the localization of ZO-1 and E-cadherin (E-Cad), which are tight-junction and adherens-junction markers, respectively, along with that of Cldn6. As expected, ZO-1 and E-Cad, but no Cldn6 signals, were localized in a zipper-like pattern at premature cell-cell junctions of control F9 cells (Figure 1C). In sharp contrast, these markers were linearly concentrated along cell borders in differentiated F9:Cldn6 cells. Surprisingly, Cldn6 dose-dependently elevated mRNA and protein levels of several other tight-junction molecules including Cldn7 [28], occludin (Ocln) [29] and ZO-1α+ variant [30] in F9 cells (Figure 1D, 1E). On the other hand, expression amounts of Cldn4 in F9 cells were decreased by Cldn6 in a dose-dependent manner (Figure 1D, 1E). Double immunostaining analysis showed that Cldn7, Ocln, ZO-1, and ZO-1α+ variant were colocalized with Cldn6 at the apical-most tips of lateral membranes of F9:Cldn6 cells, to form beltlike tight junctions, and that Cldn7 and Ocln were recruited to a part of Cldn6-positive immature cell-cell junctions (Figure 2A, 2B; and data not shown). By contrast, E-Cad was distributed along entire lateral membranes in these cells, and Cldn4 was not observed along cell-cell boundaries in general but in the cytoplasm (Figure 2A, 2C). Moreover, by freeze-fracture electron microscopy, tight-junction strands composed of anastomosing dots were detected in F9:Cldn6 cells but not in control F9 cells (Figure 3A; and data not shown).

Since microvilli are generated on the apical surfaces of epithelia as a landmark for apicobasal cell polarity, we next determined, by scanning electron microscopy, whether microvillus formation was induced in F9:Cldn6 cells. In their differentiated areas, modest amounts of microvilli were evident at the apical plasma membranes and along cell boundaries, whereas filopodia- and lamellipodia-like structures, but few microvilli, were detected in control F9 cells (Figure 3B). Consistent with these data, microvillus components such as ezrin, radixin, and ezrin/radixin/moesin-binding phosphoprotein-50 (EBP50) were apically enriched in F9:Cldn6 and Dox-treated F9 L32T2:Cldn6 cells (Figure 2D). Note however that the microvillus constituents were not concentrated on the apical surfaces of undifferentiated cells possessing Cldn6-positive premature cell-cell junctions (Figure 2D). In addition, the expression of EBP50 was clearly induced in these cells, while that of ezrin and radixin was marginally altered (Figure 1D, 1E), as in F9 L32T2:HNF4α [19].

**Figure 4:**
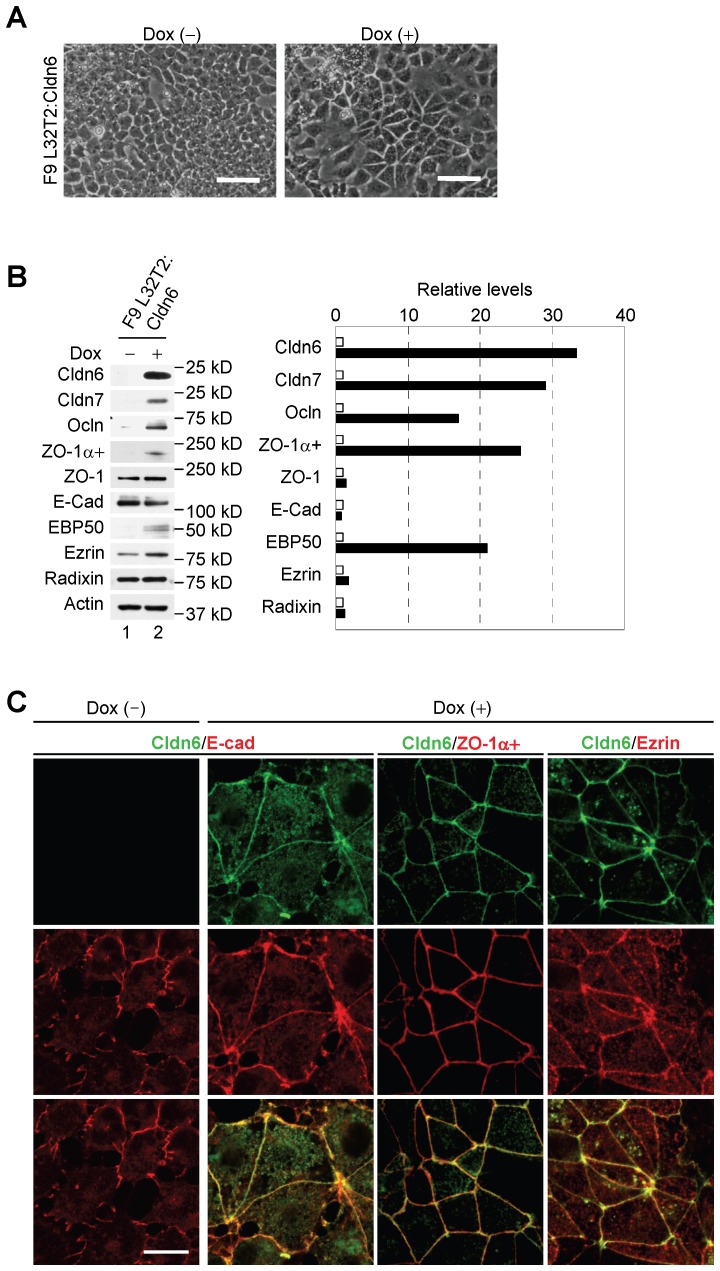
Conditional expression of Cldn6 provokes epithelial differentiation in F9 stem cells. (A) Morphological appearance of F9 L32T2:Cldn6 cells exposed for 96 h to the vehicle or 1.0 µg/ml doxycycline (Dox). Scale bars, 50 µm. (B) Western blot for the indicated proteins in F9 L32T2:Cldn6 cells treated for 48 h with the vehicle or 1.0 µg/ml Dox. Quantification of the protein levels is shown in the histograms. (C) Double immunostaining for the indicated proteins in F9 L32T2:Cldn6 cells with or without 1.0 µg/ml Dox stimulation. Scale bar, 20 µm.

Similar epithelial morphology was observed in F9 L32T2:Cldn6 cells, which conditionally expressed Cldn6, after 96 h of 1 µg/ml doxycycline (Dox) treatment (Figure 4A). Moreover, induction of Cldn7, Ocln, ZO-1α+ and EBP50, as well as the formation of mature cell-cell junction and cell polarity, were also detected in Dox-exposed F9 L32T2:Cldn6 cells (Figure 4B, 4C). Thus, Cldn6 expression appeared to trigger the establishment of mature cell-cell junctions and apicobasal cell polarity in F9 stem cells.

### Cldn6-triggered Epithelial Morphogenesis Depends on its Subcellular Localization

**Figure 5:**
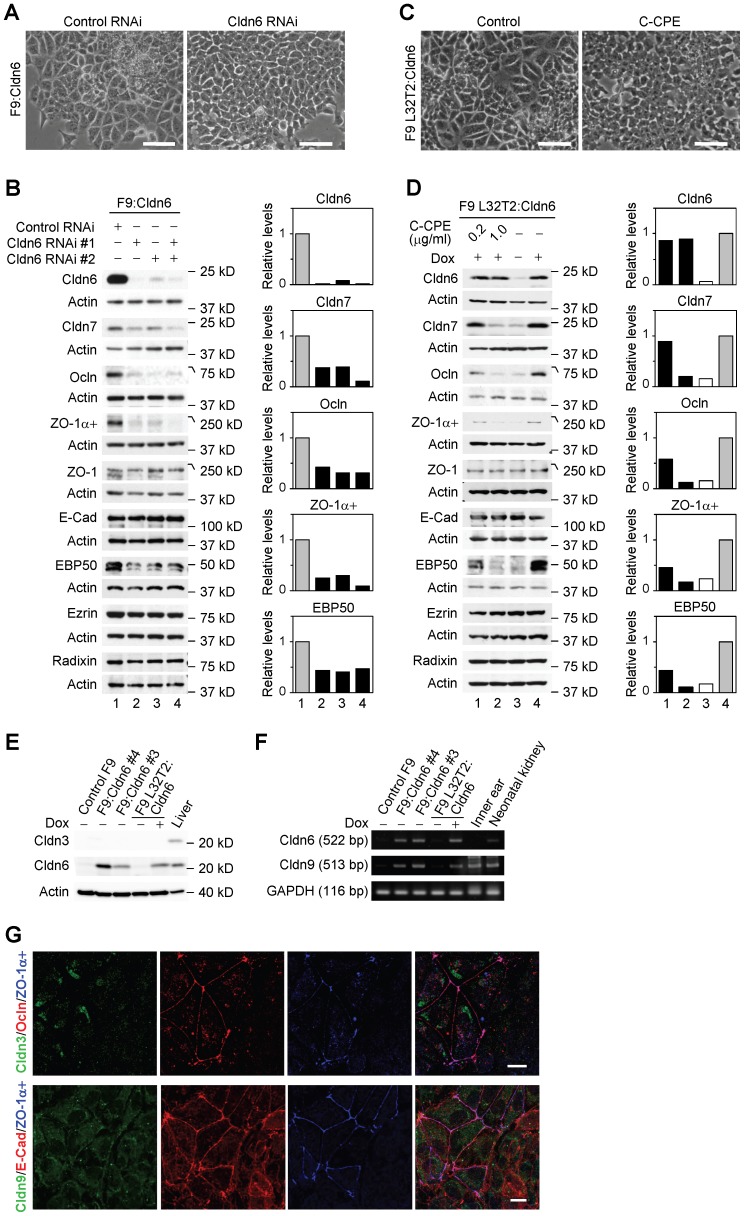
Cldn6 knockdown and C-CPE treatment prevent Cldn6-induced epithelial differentiation in F9 stem cells. (A and C) Inhibitory effects of Cldn6 knockdown and C-CPE (1.0 µg/ml) on morphological appearance of F9:Cldn6 and doxycycline (Dox)-exposed F9 L32T2:Cldn6 cells. Scale bars, 50 µm. (B) Western blot for the indicated proteins in F9:Cldn6 cells transfected with negative control siRNA or two distinct siRNAs against Cldn6 (#1, #2, or both). Quantification of the protein levels is shown in the histograms. (D) Western blot for the indicated proteins in F9 L32T2:Cldn6 cells grown for 96 h with the vehicle, 1.0 µg/ml Dox alone, or together with 0.2 and 1.0 µg/ml C-CPE. (E and F) Western blot (E) and RT-PCR (F) for the indicated molecules in control F9, F9:Cldn6, F9 L32T2:Cldn6 cells, and the mouse tissues. F9 L32T2:Cldn6 cells were cultured for 72 h with either the vehicle or 1.0 µg/ml Dox. (G) Confocal images of F9:Cldn6 cells stained for either Cldn3 together with Ocln and ZO-1α+ or Cldn9 with and E-cad ZO-1α+. Scale bar, 20 µm.

**Figure 6:**
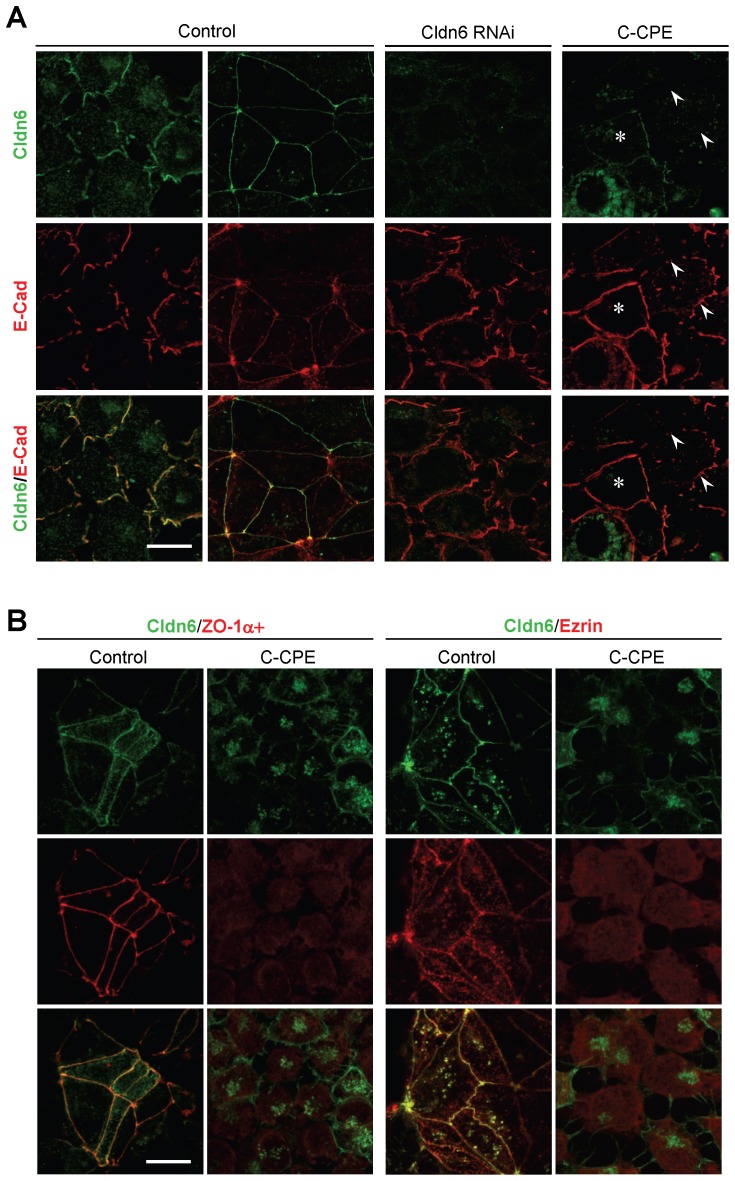
Cldn6 suppression and C-CPE exposure reduce Cldn6-induced epithelial morphogenesis. (A) Confocal images of F9 L32T2:Cldn6 cells stained for Cldn6 with E-Cad. The cells grown for 96 h in the presence of 1.0 µg/ml doxycycline (Dox) were treated with the vehicle, siRNA against Cldn6, or 1.0 µg/ml C-CPE. Arrowheads show CPE-induced disappearance of Cldn6 from cell junctions, and the asterisk indicates the residual Cldn6 signals along cell borders. Scale bar, 20 µm. (B) Confocal images of F9 L32T2:Cldn6 cells stained for Cldn6 together with the tight-junction marker ZO-1α+, and the apical marker ezrin. Dox-exposed cells were treated with the vehicle or 1.0 µg/ml C-CPE. Scale bar, 20 µm.

**Figure 7:**
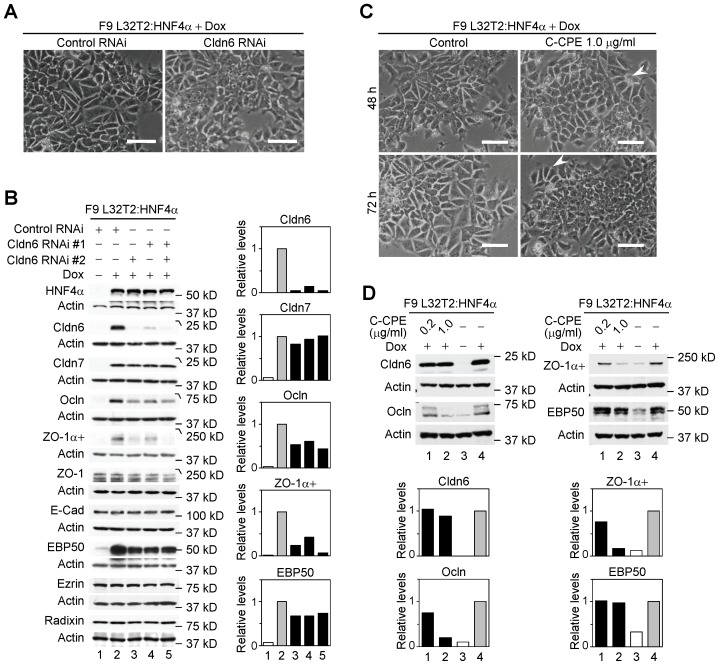
Cldn6 suppression and C-CPE exposure decrease HNF4α-induced epithelial morphogenesis. (A) Effect of Cldn6 knockdown on morphological appearance of F9 L32T2:HNF4α cells. The cells were transfected with negative control siRNA or siRNAs against Cldn6 (#1 and #2), and grown for 96 h with 1.0 µg/ml doxycycline (Dox). Scale bars, 50 µm. (B) Western blot for the indicated proteins in F9 L32T2:HNF4α cells transfected with negative control siRNA or two distinct siRNAs against Cldn6 (#1, #2, or both), and grown for 72 h in the presence or absence of 1.0 µg/ml Dox. Quantification of the protein levels is shown in the histograms. (C) Effect of C-CPE on morphological appearance of F9 L32T2:HNF4α cells. The cells were cultured for 48 h and 72 h in the presence of 1.0 µg/ml Dox together with the vehicle or 1.0 µg/ml C-CPE. The arrowhead indicates residual differentiated cells. Scale bars, 50 µm. (D) Western blot for the indicated proteins in F9 L32T2:HNF4α cells grown for 72 h in the presence or absence of 1.0 µg/ml Dox together with the vehicle, 0.2 or 1.0 µg/ml C-CPE.

Cldn6 knockdown remarkably suppressed morphological differentiation and cell-junction formation in F9:Cldn6 and F9 L32T2:Cldn6 cells (Figure 5A, 6A). In addition, Cldn6 suppression decreased upregulation of Cldn7, Ocln, ZO-1α+, and EBP50 expression in F9:Cldn6 cells (Figure 5B). We then utilized the C-terminal half of *Clostridium Perfringens* enterotoxin (C-CPE), since it binds to the second extracellular domain of Cldn3, Cldn4, Cldn6 and Cldn9 with high affinity, leading to their exclusion from tight junctions with no obvious cell damage [31]–[34]. When Dox-exposed F9 L32T2:Cldn6 cells were treated with 1 µg/ml C-CPE, their morphological differentiation was almost completely blocked (Figure 5C). It is noteworthy that C-CPE exposure to F9 L32T2:Cldn6 cells eliminated Cldn6 from cell junctions without any changes in its total protein level, and did suppress expression of Cldn7, Ocln, ZO-1α+, and EBP50, as well as mature junction formation (Figure 5D, 6A). Establishment of cell junctions and polarity were also hindered in C-CPE-exposed F9:Cldn6 (Figure 6B). The effects of C-CPE were attributed to its binding to Cldn6, because Cldn3 expression was barely detected in these cells and Cldn4 and Cldn9 showed no or little cell-junctional localization (Figure 2C, 5E, 5F, 5G). Furthermore, similar inhibitory effects of either Cldn6 knockdown or C-CPE treatment were partly observed in F9 L32T2:HNF4α cells (Figure 7A, 7B, 7C, 7D), in which inducible expression of HNF4α provokes robust epithelial differentiation [17]–[19]. Thus, these results strongly suggested that the Cldn6-triggered epithelial morphogenesis in F9 stem cells depended not only on its expression amount but also on the subcellular localization, and that its signal could be transmitted at cell-cell junctions.

### Cldn6 Contributes to Epithelial Morphogenesis in Mouse Embryoid Bodies

**Figure 8:**
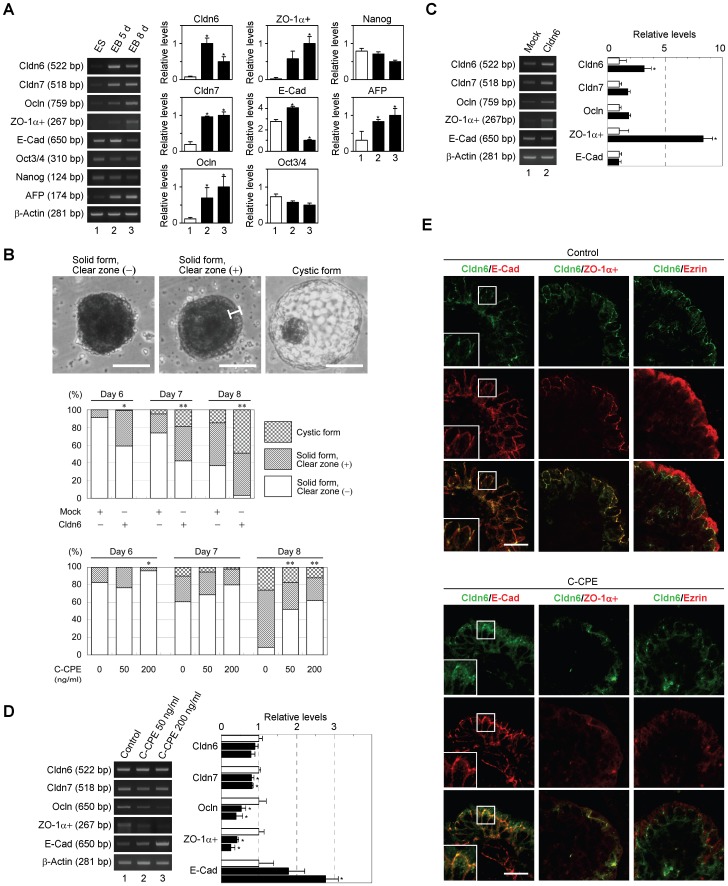
Effects of Cldn6 overexpression and C-CPE treatment on epithelial differentiation in mouse embryoid bodies. (A) RT-PCR for the indicated molecules in mouse embryonal stem (ES) cells and embryoid bodies (EBs). EBs were grown in 5-d and 8-d suspension culture. Quantification of the mRNA levels is shown in the histograms (mean+SD; *n* = 4). *P<0.05 compared with values of ES cells. (B) Top, the three distinct forms of the appearance of EBs. The bracket indicates the clear zone, which corresponds to the endoderm cell layer. Scale bars, 100 µm. Middle, embryonic stem cells were transfected with the mock or Cldn6 expression vectors, and EBs were cultured for 6–8 days, followed by categorization of their morphological appearance. Bottom, EBs were cultured for 6–8 days with the vehicle, 50 or 200 ng/ml C-CPE, and their appearance was classified. In statistical analyses, all values were compared with control values. *P<0.05; **P<0.001. (C and D) RT-PCR for the indicated molecules in the mock- and Cldn6-transfected 8-d EBs, and those treated with the vehicle or C-CPE. Quantification of the mRNA levels is shown in the histograms (mean+SD; n = 4). *P<0.05. (E) Confocal images of 8-d EBs stained for Cldn6 together with the basolateral marker E-cadherin (E-Cad), the tight-junction marker ZO-1α+, and the apical marker ezrin. EBs were grown for 8 days in the presence or absence of 200 ng/ml C-CPE. Scale bars, 50 µm.

As previously reported [35], [36], when embryonic stem (ES) cells were grown in suspension, they aggregated to form embryoid bodies (EBs) that were surrounded by the outermost layer of epithelial cells (visceral endoderm-like cells), and expressed α-fetoprotein and several tight-junction markers (Figure 8A, 8B; top). Therefore, we used EBs to further support the importance of Cldn6 in epithelial morphogenesis. As expected, overexpression of Cldn6 led to a significant increase in the proportion of EBs delineated by epithelia (solid form with clear zone, and cystic form) at 6, 7, and 8 days of suspension culture (Figure 8B; middle). In Cldn6-transfected EBs, mRNA levels of Cldn7, Ocln, and ZO-1α+ were elevated compared with those in mock-transfected EBs (Figure 8C). Conversely, 50 and 200 ng/ml of C-CPE decreased the ratio of epithelia-covered EBs and expression of Cldn7, Ocln, and ZO-1α+ transcripts with no obvious alteration in the expression level of Cldn6 (Figure 8B; bottom, 8D). E-Cad expression in EBs was significantly diminished and enhanced during the culture period and upon C-CPE treatment, respectively (Figure 8A, 8D), both of which may reflect the extent of the stemness, because E-cad also likely acts as a pluripotent factor [37]. Furthermore, C-CPE exposure to EBs occasionally inhibited the concentration of Cldn6 in cell-cell junctions of residual epithelia, and markedly reduced ZO-1α+ and ezrin signals (Figure 8E). Hence, these results further strengthened the functional relevance of Cldn6 signaling in epithelial morphogenesis.

Collectively, our study highlighted that the single cell-adhesion molecule Cldn6 was enough to induce establishment of epithelial cell junctions and polarity in F9 and embryonal stem cells. This conclusion was drawn from results using stable or conditional expression and RNAi strategies in these cells. Cldns, the backbone of tight junctions, create paracellular barriers and pores controlling molecular penetration of ions, solutes, water, and cells [22]–[24], [38]. We have demonstrated that at least the selective Cldn also plays a fundamental role in the decision of stem cell fate, namely epithelial differentiation. When F9:Cldn6 and Dox-exposed F9 L32T2:Cldn6 were grown in suspension as aggregates, the outermost layer of cells remarkably differentiated into visceral endoderm-like cells (our unpublished results), further supporting our conclusion. Since Cldn6 is expressed in a wide variety of embryonal epithelia such as the trophectoderm, visceral endoderm, epiblast, hypoblast, and definitive endoderm, as well as embryonic epithelia of the entire gut and lung [27], [32], Cldn6 may also control differentiation from stem cells to these epithelia. On the other hand, Cldn6-null mice exhibit no abnormality, suggesting that other Cldns may compensate for the function of Cldn6 *in vivo* [27]. Along this line, it is interesting to note that “Cldn-low subtype”, which shows high expression of stem cell-like and mesenchymal markers and low expression of epithelial markers including Cldn3, Cldn4, and Cldn7, is identified from both breast cancers and normal breast tissues [39], [40]. Moreover, the forced expression of Cldn7 in F9 stem cells resulted in weak induction of Cldn6 expression and very limited epithelial morphogenesis (our unpublished results), indicating that epithelial differentiation may be distinctively triggered by Cldn-subtype.

We previously demonstrated that several tight-junction proteins ZO-1, ZO-2, ZO-3, junctional adhesion molecule (JAM)-B and JAM-C, as well as cell polarity proteins PAR-3, PAR-6 and atypical protein kinase C, were accumulated to E-Cad/nectins-positive primordial cell junctions in F9 cells (18). Taking together with present study using C-CPE-treated F9 and ES cells, we conclude that recruitment of Cldn6 to premature intercellular junctions is sufficient to induction of Cldn7, Ocln, ZO-1α+ and EBP50 expression, as well as the formation of developed cell-cell junction and cell polarity. In conclusion, we have found that Cldn6-adhesion signaling provokes epithelial differentiation from stem cells. It should be determined how Cldn6 signal for epithelial morphogenesis is mediated in future experiments.

## Materials and Methods

### Antibodies

The following primary antibodies were used: rabbit anti-Cldn3 (1∶100 for immunohistochemistry [IHC], 1∶1,000 for immunoblot [IB]; Invitrogen, 34–1700), mouse anti-Cldn4 (1∶100 for IHC, 1∶500 for IB; Invitrogen, 32–9400) rabbit anti-Cldn6 (1∶200 for IHC, 1∶2,000 for IB; Immuno-Biological Laboratories, 18865) [18], goat anti-Cldn6 (1∶100 for IHC; Santa Cruz Technology, sc17669), rabbit anti-Cldn7 (1∶100 for IHC, 1∶1,000 for IB; Immuno-Biological Laboratories, 18875) [18], rabbit anti-Cldn9 (1∶100 for IHC, 1∶1,000 for IB; Santa Cruz Technology, sc17672), rabbit anti-Ocln (1∶1,000 for IB; LifeSpan BioScience, LS-B2187), mouse anti-Ocln (1∶100 for IHC; Invitrogen, 33–1500), rat anti-ZO-1 (detecting ZO-1α+ variant, 1∶100 for IHC, 1∶1,000 for IB; Santa Cruz Technology, sc33725), rabbit anti ZO-1 (1∶100 for IHC, 1∶1,000 for IB; Invitrogen, 61–7300), mouse anti-E-cadherin (1∶200 for IHC, 1∶2,000 for IB; Becton Dickinson Bioscience, 610182), goat anti-HNF4α (1∶1,000 for IB; Santa Cruz Technology, sc6556), rabbit anti-EBP50 (1∶100 for IHC, 1∶2,000 for IB; Affinity Bio Reagents, PA-1-090), rat anti-ezrin (1∶200 for IHC, 1∶2,000 for IB; Sanko Junyaku, AB01006), rat anti-radixin (1∶200 for IHC, 1∶2,000 for IB; Sanko Junyaku, AB01007), and rabbit anti-actin (1∶4,000 for IB; Sigma-Aldrich, A2103). The secondary antibodies used were as follows: HRP-conjugated anti-mouse, -rat, and -rabbit IgG (Dako Cytomation), AlexaFluor488 (green)-labeled anti-mouse, -rabbit, and -goat IgG (Invitrogen), AlexaFluor594 (red)-labeled anti-mouse, -rat, -rabbit, and -goat IgG (Invitrogen).

### Cell Culture, Plasmids and Cell Lines

F9 L32T2 and F9 L32T2∶4α cells were generated as described previously [15], [17]. To establish F9:Cldn6 and F9 L32T2:Cldn6 cell lines, F9 L32T2 cells were electroporated with the expression vectors pD402A-KmCldn6 [18] and pTRE-KmCldn6, respectively, together with the puromycin-resistant gene expression vector pHRL-puro1 [41]. Cells were plated in DMEM with 10% FBS, and the medium was changed every 2 d. They were treated with doxycycline (Dox) and C-CPE 1 d after plating.

Mouse ES cells were provided from RIKEN BioResource Center Cell Bank (Cell No. AES0008). They were cultured on SNL 76/7 feeder cells (SIGMA 070328001) in DMEM (high glucose and 1 mM sodium pyruvate) supplemented with 15% KnockOut serum replacement (Gibco), 0.1 mM 2-mercaptoethanol (Sigma), 1,000 U/ml leukemia inhibitory factor (Wako), 0.1 mM nonessential amino acids (Gibco), and GlutaMAX solution (Gibco).

The morphology of these cells was checked by phase-contrast microscopy (Axiovert 200 and HAL100; Carl Zeiss).

### Embryoid Formation

For embryoid body (EB) formation, 6×10^6^ ES cells were first cultured on gelatin-coated 10 cm-culture dishes without feeder cells for 3 d in the same medium, and subjected to suspension culture in three 10 cm-bacterial dishes in the DMEM with 15% FBS, 0.1 mM 2-mercaptoethanol (Sigma), 0.1 mM nonessential amino acids (Gibco), and GlutaMAX solution (Gibco). The medium was changed every other day. C-CPE was added 1 d after being shifted to the suspension culture. For transient expression of Cldn6 in ES cells, cells were treated with supernatant produced by Lenti-X Expression System (Clontech) and the expression vector pLVSIN-EF1α-neo-KmCldn6 at 1 d after plating without feeder cells according to the manufacture’s method. Two hundred EBs at 6–8 d in each dish were classified into three categories, namely solid form without clear zone, solid form with clear zone, and cystic form.

### RNAi and Transfection

Stealth Select siRNAs were synthesized by Invitrogen. The sequences were as follows: Cldn6 RNAi #1, sense (5′-AAGAUUUGCAGACCAGUAGAGGCCA-3′) and antisense (5′-UGGCCUCUACUGGUCUGCAAAUCUU-3′); Cldn6 RNAi #2, sense (5′-CCCACUCUAUCAUCCAGGACUUCUA-3′) and antisense (5′-UAGAAGUCCUGGAUGAUAGAGUGGG-3′). Stealth RNAi negative control kit (Invitrogen) was used for control RNAi. Cells were transfected with siRNAs by using Lipofectamine RNAi MAX reagent (Invitrogen) according to the manufacture’s protocol.

### C-CPE Construction and Purification

The expression vector pET16b coding C-CPE194-319 [42] was kindly provided by Dr. M. Kondoh (Osaka University) [43]. E. coli BL21 (DE3) transformed with the vector was grown at room temperature, and protein expression was stimulated by the addition of isopropyl-1-thio-b-D-galactoside, followed by purification using TALON His purification resin (Clontech).

### Immunoblot

Immunoblot analysis was performed as previously described [19], [44]. Can Get Signal Immunoreaction Enhancer Solution kit (TOYOBO) was used for the detection of Cldn4 and ZO-1α+. Each blot was stripped with Restore Western blot stripping buffer (Pierce Chemical) and immunoprobed with anti-actin antibody. Signals in immunoblots were quantified using Image-J software (Wayne Rasband National Institutes of Health). The protein levels were normalized to the corresponding actin levels, and their relative levels were presented.

### Immunohistochemistry and Electron Microscopy

F9 cells were grown on rat tail collagen-coated coverslips. EBs were concentrated in conical tubes by gravity flow and embedded in NEG50 compound (Richard-Allan Scientific). After being frozen, they were subjected to an 8 µm thin slice. These samples were fixed in a 1∶1 acetone/ethanol solution for 10 min at −20°C. After washing with PBS, the samples were preincubated in PBS containing 5% skim milk. They were subsequently incubated overnight at 4°C with primary antibodies in PBS and rinsed again with PBS, followed by a reaction for 1 h at room temperature with appropriate secondary antibodies. All samples were examined using a laser-scanning confocal microscope (LSM510 with Axiovert 100 M; Carl Zeiss) and a planApo 60×NA 1.40 oil immersion objective (Nikon). Photographs were processed with Photoshop 6.0 (Adobe), and processed with Photoshop 6.0 (Adobe).

Scanning electron microscopy and freeze-fracture electron microscopy were performed as previously described [16], [19].

### RT-PCR Analysis

For analysis of gene expression, total RNA was isolated from cells and tissues using RNeasy (QIAGEN), and RT-PCR was performed using Superscript II RT Kit (Life Technology). The PCR primers for mouse cDNAs were as follows: ZO-1α+ variant (GenBank accession no. NM_009386), sense (5′-CCTGGACTTAAGCCAGC-3′) and antisense (5′-CCTTCCTGRACACCTTTGC-3′); ZO-1 (GenBank accession no. NM_009386), sense (5′-CATAGAATAGACTCCCCTGG-3′) and antisense (5′-GCTTGAGGACTCATACCTGT-3′); E-cadherin (GenBank accession no. NM_009864), sense (5′-CGTGATGAAGGTCTCAGCC-3′) and antisense (5′-ATGGGGGCTTCATTCAC-3′); HNF4α (GenBank accession no. NM_008261), sense (5′-AGTGCCCTGTGTGCCATCTGTG-3′) and antisense (5′-AGATGATGGCTTTGAGGCAGGCG-3′); Cldn9 (GenBank accession no. NM_020293.3), sense (5′-CTTTCATCGGCAACAGCATCG-3′) and antisense (5′-TCTTATCCAGTCCCGAAGCAC-3′); AFP (GenBank accession no. NM_007423), sense (5′-TCGTATTCCAACAGGAGG-3′) and antisense (5′-AGGCTTTTGCTTCACCAG-3′); Oct-4 (also known as Pou5f1; GenBank accession no. NM_013633), sense (5′-GTTCTCTTTGGAAAGGTGTTC-3′) and antisense (5′-CTCGAACCACATCCTTCTCT-3′); Nanog (GenBank accession no. NM_028016), sense (5′-GTACCTCAGCCTCCAGCA-3′) and antisense (5′-CAACCACTGGTTTTTCTGC-3′); β-Actin (GenBank accession no. NM_007393), sense (5′-GGCATTTGTTACCAACTGGGAC-3′) and antisense (5′-AGGCTTTTGCTTCACCAG-3′); GAGDH (GenBank accession no. NM_008084.2), sense (5′-AACCACGAGAAATATGACAACTCACT-3′) and antisense (5′AGCCCTTCCACAATGCCAAAG-3′). The primers for Cldn4, Cldn6, Cldn7, Ocln, 36B4, EBP50, Ezrin, and Radixin were previously described [16], [19]. Each cycle number was as follows; Cldn6∶ 18–20 and 24–26 for F9 and ES cells, respectively, Cldn7∶ 28–30, Ocln: 32–34, ZO-1α+: 33–35, ZO-1∶ 29–31, E-cadherin: 26–28, EBP50∶ 25–27, ezrin: 30–32, radixin: 29–31, HNF4α: 28–30, AFP: 28–30, Oct-4∶ 30–32, Nanog: 30–32, 36B4∶ 16–18, and β-Actin: 22–24. Aliquots of PCR products were loaded onto 1.0–2.0% agarose gel and analyzed after staining with ethidium bromide. Signals in RT-PCR analysis were quantified using Image-J software (Wayne Rasband National Institutes of Health).

### Animals and Tissue Preparation

Mice were anesthetized with diethyl ether, and specimens of the liver, inner ear and neonatal kidney were obtained. All aspects of the study were approved by the Animal Use and Care Committee of Fukushima Medical University School of Medicine.

### Statistical Analysis

The values of RT-PCR are presented as the mean+SD. Original values were divided by the corresponding 36B4 or β-actin signal level, and mean and SD values were calculated from 4 samples. The values were divided by the mean values, and their relative levels were analyzed by paired sample two-tailed *t*-test to evaluate statistical significances. The proportion of categorized EBs in each group was subjected to chi-squared test, and their P values were calculated.
